# Neonicotinoids in bees: a review on concentrations, side-effects and risk assessment

**DOI:** 10.1007/s10646-012-0863-x

**Published:** 2012-02-18

**Authors:** Tjeerd Blacquière, Guy Smagghe, Cornelis A. M. van Gestel, Veerle Mommaerts

**Affiliations:** 1 Plant Research International, Wageningen University & Research, PO Box 69, 6700 AB Wageningen, The Netherlands; 2 Department of Crop Protection, Faculty of Bioscience Engineering, Ghent University, Coupure Links 653, 9000 Ghent, Belgium; 3 Department of Animal Ecology, Faculty of Earth and Life Sciences, VU University, De Boelelaan 1085, 1081 HV Amsterdam, The Netherlands

**Keywords:** Honey bee, Bumble bee, Solitary bee, Lethal toxicity, Sublethal effects, Reproduction, Behavioral effect, Risk assessment, Neonicotinoids, Residues

## Abstract

Neonicotinoid insecticides are successfully applied to control pests in a variety of agricultural crops; however, they may not only affect pest insects but also non-target organisms such as pollinators. This review summarizes, for the first time, 15 years of research on the hazards of neonicotinoids to bees including honey bees, bumble bees and solitary bees. The focus of the paper is on three different key aspects determining the risks of neonicotinoid field concentrations for bee populations: (1) the environmental neonicotinoid residue levels in plants, bees and bee products in relation to pesticide application, (2) the reported side-effects with special attention for sublethal effects, and (3) the usefulness for the evaluation of neonicotinoids of an already existing risk assessment scheme for systemic compounds. Although environmental residue levels of neonicotinoids were found to be lower than acute/chronic toxicity levels, there is still a lack of reliable data as most analyses were conducted near the detection limit and for only few crops. Many laboratory studies described lethal and sublethal effects of neonicotinoids on the foraging behavior, and learning and memory abilities of bees, while no effects were observed in field studies at field-realistic dosages. The proposed risk assessment scheme for systemic compounds was shown to be applicable to assess the risk for side-effects of neonicotinoids as it considers the effect on different life stages and different levels of biological organization (organism versus colony). Future research studies should be conducted with field-realistic concentrations, relevant exposure and evaluation durations. Molecular markers may be used to improve risk assessment by a better understanding of the mode of action (interaction with receptors) of neonicotinoids in bees leading to the identification of environmentally safer compounds.

## Introduction

Bees, including honey bees, bumble bees and solitary bees, are the prominent and economically most important group of pollinators worldwide; 35% of the world food crop production depends on pollinators (Klein et al. 2007; Velthuis and van Doorn 2006), accounting for an annual value of 153 billion Euros (Gallai et al. 2009). In Europe, for instance, the production of 84% of crop species is to some extent depending on animal pollination (Williams 1994). Bees also provide important pollination services to wild plants, of which in Europe 80% need insects for pollination (Kwak et al. 1998), so confirming their ecological importance. The decline of pollinating species, which has grown over the last decades, may lead to a parallel decrease of plant species, or vice versa (Biesmeijer et al. 2006; National Research Council of the National Academies 2007; Goulson et al. 2008). More specifically, there is a great concern about the decline of the honey bee (*Apis mellifera*) in several parts of the world (Oldroyd 2007; Stokstad 2007; VanEngelsdorp and Meixner 2010). It is now accepted that the abundance of pollinators in the environment is influenced by multiple factors, including biotic ones like pathogens, parasites, availability of resources due to habitat fragmentation and loss; and abiotic ones like climate change and pollutants (Decourtye et al. 2010; Neumann and Carreck 2010; Kluser et al. 2011). Although the putative causes are still currently analyzed, the extensive use of chemical pesticides against pest insects for crop protection may have contributed to the loss of pollinators.

To feed the fast growing global population, chemical insecticides are important to crop productivity in intensive farming systems where they preserve about one-fifth of the crop yield (Oerke and Dehne 2004). Good examples are the major staple crops like cereals, soybeans, maize, and many fruit and vegetable crops. Within the different insecticide classes, the neonicotinoid insecticides, which include imidacloprid, acetamiprid, clothianidin, thiamethoxam, thiacloprid, dinotefuran and nitenpyram, are an important group of neurotoxins specifically acting as antagonists of the insect nicotinic acetylcholine receptors (nAChR) (Matsuda et al. 2001; Elbert et al. 2008). Since the introduction of imidacloprid in the early 1990s, the use of different neonicotinoid insecticides has grown considerably. They are used extensively for the control of important agricultural crop pests by spraying and also widely used in seed dressings and soil additions. In the latter two cases residues of these systemic insecticides can be present at ‘trace’ levels in the plant pollen and nectar. So potentially, bees could be exposed at a large scale to insecticide residues originating from crop seed dressings.

To date in the international scientific literature >100 papers appeared with the keywords “neonicotinoids/imidacloprid” and “bee”, the first being published in 1992, and an impressive cumulative number of citations near to 1,500. In addition many reports have appeared in different types of the public media, highlighting the awareness by the different stakeholders in the field related to pesticides, bees, environment, toxicology, pollination and agriculture.

This review gives, for the first time, a summary of the data published over the last 15 years on concentrations of neonicotinoid insecticides recovered in plants and bees and their products. This analysis of the literature took into consideration the different crops, the methods of application and the importance of metabolism, and covered data from different countries and continents. Second, the publicly available data on side-effects of the different neonicotinoid insecticides towards honey bees, bumble bees and other bee species are summarized, and critically analyzed with a special emphasis on sublethal effects on reproduction, foraging behavior, memory/learning abilities and overwintering success. A third part focuses on the potential applicability of the new stepwise risk assessment scheme as proposed for systemic pesticides (Alix et al. 2009; Thompson 2010), for more adequately assessing risks for side-effects by neonicotinoid insecticides. The latter assessment took into account the characteristics of doses of neonicotinoid insecticides in their field-realistic range and followed the classical tiered approach from the laboratory to field-related conditions and from exposure of individual bees to the colony level. The importance of the use of adults and larvae (brood) together with the scoring of lethal and sublethal biological endpoints is also discussed. Points of comparison and experimental advantages and difficulties between honey bees, bumble bees and other bees are discussed. Attention is paid to the use of mixtures containing neonicotinoid insecticides that can synergize their hazards for bees. Our paper concludes with some targets for research and recommendations for future risk assessment studies, specifically with the aim to assess the global bee colony health status.

## Concentrations and metabolism of neonicotinoid insecticides in plants and bees in relation to pesticide application

### Translocation of residues in plants, nectar and pollen

Several studies have examined the translocation of imidacloprid from seed treatment to different parts of sunflower (*Helianthus annuus*) plants. In a greenhouse experiment with sunflowers treated with 0.7 mg ^14^C-imidacloprid per seed (Gaucho WS, 700 g kg^−1^) average imidacloprid concentrations amounted 3.9 ± 1.0 μg kg^−1^ in pollen and 1.9 ± 1.0 μg kg^−1^ in nectar (Schmuck et al. 2001). Nectar contained only imidacloprid and in pollen 85% of the ^14^C-residues were present as imidacloprid (no metabolites were detected). In a field study at the dosage of 1 mg per seed (i.e. 30% higher than the recommended dose) no imidacloprid or metabolites were found in nectar and pollen, while the leaves of the sunflowers contained imidacloprid at 7 μg kg^−1^ and the hydroxy-metabolite at <5 μg kg^−1^ (Schmuck et al. 2001). Only 5% of the ^14^C-imidacloprid dose (1 mg per seed) was taken up from the seed after 4 weeks of sunflower growth in a climate-controlled cabinet. At flowering 90% of the dose was estimated to be still present in the soil. In the plant leaves mainly imidacloprid (approximately 50% of total ^14^C) was found together with three metabolites (30–50% of ^14^C). Imidacloprid concentrations decreased from the first leaves to the top leaves; levels in sunflower pollen were <0.5–36 μg kg^−1^ (Laurent and Rathahao 2003). Sunflower plants showed decreasing imidacloprid levels with time till the moment of capitule (flower head of Asteraceae) formation, but thereafter concentrations increased again. Imidacloprid concentrations in plants differed between sunflower varieties with average concentrations in the flowers between 5 and 10 μg kg^−1^ (Bonmatin et al. 2003). The latter study also determined imidacloprid residues in pollen samples of maize and sunflower that received a seed treatment. In 58% of the pollen samples imidacloprid was found with an average concentration of 3 μg kg^−1^ (range 1–11 μg kg^−1^) for sunflower. In 80% of the maize pollen samples imidacloprid was found at an average concentration of 2 μg kg^−1^ (5 samples only; range 1–3 μg kg^−1^) (Bonmatin et al. 2003), while a follow-up of this study reported an average concentration of 3.0 μg kg^−1^ (Charvet et al. 2004).

When sunflower and maize (without seed treatment) were planted on soils still containing imidacloprid at 2–18 μg kg^−1^ from earlier treatments, no imidacloprid was detected in pollen and nectar (Schmuck et al. 2001; Charvet et al. 2004).

Girolami et al. (2009) found that part of the imidacloprid taken up by maize seedlings can be eliminated through the guttation fluid, i.e. the droplets on the leaf tip. Excretion of guttation fluid seems limited to the first 3 weeks after germination (Girolami et al. 2009; Thompson 2010) and is affected by humidity, temperature, growth stage, water stress, root depth and soil water potential (Tapparo et al. 2011). During the first 3 weeks after emergence, imidacloprid concentrations can be very high. From a seed treatment of 0.5 mg per seed (Gaucho 350 FS), the imidacloprid concentrations in the guttation fluid of plants grown in the laboratory ranged between 47 ± 9.9 and 83.8 ± 14.1 mg l^−1^ (Girolami et al. 2009). Similarly, residues of clothianidin (23.3 ± 4.2 mg l^−1^ from plants treated with 1.25 mg per seed as Poncho) and thiamethoxam (11.9 ± 3.32 mg l^−1^; 1 mg per seed as Cruiser 350 FS) were found in the guttation fluid (Girolami et al. 2009). Tapparo et al. (2011) reported a decline of imidacloprid concentrations in the guttation fluid of maize plants that were dosed at 0.5 mg per seed (Gaucho) and grown in the greenhouse, from 80.1 mg l^−1^ after 1 day to 17.3 mg l^−1^ after 8–10 days, but the concentrations increased again to 60.1 mg l^−1^ during the next 10 days. At a dose of 1.25 mg per seed, imidacloprid concentrations in guttation drops that were collected during the first 6 days after emergence at the top of the leaves, ranged between 103 and 346 mg l^−1^, while at the crown they amounted 8.2–120 mg l^−1^. In the guttation fluid collected from plants grown in the field during the first day after emergence, imidacloprid concentrations ranged between 77 and 222 mg l^−1^ (Tapparo et al. 2011). Similar patterns were also seen for clothianidin (7.3–102 mg l^−1^) and thiamethoxam (2.9–40.8 mg l^−1^) (Tapparo et al. 2011). Thiamethoxam concentrations in guttation fluid increased with decreasing soil moisture content, from 14 to 155 mg l^−1^ for plants grown under wet conditions to 34–1,154 mg l^−1^ under dry conditions (Tapparo et al. 2011). The guttation fluid from plants growing on a field next to a plot planted with clothianidin-treated maize seeds (1.25 mg per seed; Poncho) always contained <30 μg l^−1^ clothianidin (Marzaro et al. 2011).

### Residues in bee-collected pollen, bees, honey and wax

Neonicotinoid residues in plants and plant parts only become of importance for bees once they are exposed. The most relevant measures of exposure are the concentrations in bee-collected plant materials, such as pollen, bee products like bee bread, honey and beeswax, and in the bees themselves. Table 1 summarizes reports on neonicotinoid insecticide concentrations in bee-related products as published in the literature.

**Table 1:** Overview of literature data on neonicotinoid residues in bee-collected pollen, honey and bees

Substrate	Country/area/land use/ecosystem	Dominant crop/plant species	Experimental design and statistical analysis	Chemical	No. of samples	% Samples positive	Concentration (μg kg^−1^ fresh weight)		Notes	References
							Mean^a^	Range in positive samples		
Pollen	France; 4 agricultural + 1 natural region	No data	24 Sites, 120 colonies; 4× per year; Oct 2002–Sept 2005; χ^2^ analysis to compare frequencies of pesticide occurrence between years and areas	imi	185	40.5	0.9	>0.2–5.7	No significant differences between areas; frequency in 2003 sign. higher than in 2005; many other pesticides found	Chauzat et al. (2006, 2009, 2011)
	Spain, 11 different regions	Sunflower dominant in 11.5%, wild vegetation in 78.7% and mixture in 9.8% of samples	Pollen from comb cells; χ^2^ analysis to compare pesticide occurrence in unhealthy (depopulated) and asymptomatic colonies	imi	61	0	<0.4		No significant difference in pesticide residue levels between unhealthy and healthy populations; nine other pesticides detected	Higes et al. (2010)
	Spain, 17 regions	Wild vegetation in 47.8%, crops in 38.3% of samples (10.4% sunflower, 7.8% *Medicago*, 9.5% cereals)	Stored pollen from 448 + 92 spring and 397 + 84 autumn samples in 2006 + 2007, respectively; statistical analysis not applicable to imidacloprid	imi	1,021	0	<0.4		Many pesticides detected in 42% and 31% of spring and autumn samples, respectively	Bernal et al. (2010)
	USA (Florida, California, Pennsylvania or 13 states) + samples from outside USA and Canada	No data	2007/2008 Survey incl. 13 apiaries in Florida + California, 47 colonies in Pennsylvania orchards and from ‘other’ samples; no further data analysis	imi	350	2.9	3.1	<2.0–912	Many pesticides detected	Mullin et al. (2010)
				thm		0.29^b^	53.3			
				ace		3.1	1.9	<5.0–124		
				thc		5.4	1.3	<1.0–115		
	Germany, throughout the country	Main nectar flow plants: oil seed rape (*Brassica napus*; 11–37%), sunflower (*H. annuus*; 3–4%) and corn (*Zea mays*; 16–22%)	Nation-wide survey; many beekeepers; 2005 + 2006 (50 apiaries; *n* = 105) and 2007 (*n* = 110 apiaries); sampling after rapeseed flowering; no further data analysis	imi	215	0.47^b^	3		Many pesticides detected	Genersch et al. (2010)
				thc		33	–	>1.0–199		
				clo		0	<0.1			
				ace		0.93	–	>1.0		
	Guelph, Canada	*Brassica napus*	1 ha fields; treated 400 g AI kg^−1^ seed (= 32 g ha^−1^) + control; 4 bee colonies per field for 21 days; sampling at 14 day intervals; samples pooled per field and sampling day; no further data analysis	clo	No data	Few	–	<0.5–2.59 (treated); <0.5 (control)		Cutler and Scott-Dupree (2007)
Honey	France; 4 agricultural + 1 natural region	No data	24 Sites, 120 colonies; 4× per year; Oct 2002–Sept 2005; χ^2^ analysis to compare frequencies of pesticide occurrence between years and areas	imi	239	21.8	0.7	>0.3–1.8	No differences between areas or years	Chauzat et al. (2006, 2009, 2011)
	Belgium, different areas	No data	Monitoring; no further details	imi	109	4.6	<0.084			Pirard et al. (2007)
	Belgium, different areas	Maize (0.05–2.48% of crop treated)	August–October 2004 (after flowering of *Zea mays*); no further data analysis	imi	48	8.4	0.275	>0.05 <0.5		Nguyen et al. (2009)
	North-West Spain	No data	73 Apiaries; no further data analysis	imi	91	0	<2.33			Garcia-Chao et al. (2010)
				thm		0	<0.51			
	Guelph, Canada	*Brassica napus*	1 ha fields; treated 400 g AI kg^−1^ seed (= 32 g ha^−1^) +control; 4 bee colonies per field for 21-d; sampling at 14-d intervals; no further data analysis	clo	No data	Few	–	<0.5–0.93 (treated); <0.5 (control)		Cutler and Scott-Dupree (2007)
Honey bees	France; 4 agricultural + 1 natural region	No data	24 Sites, 120 colonies; 4× per year; Oct 2002–Sept 2005; χ^2^ analysis to compare frequencies of pesticide occurrence between years and areas	imi	187	11.2	1.2	>0.3–11.1	No differences between areas or years	Chauzat et al. (2006, 2009, 2011)
	Belgium, different areas	No data	Monitoring; no further details	imi	99	0	<0.1			Pirard et al. (2007)
	Belgium, different areas	Maize (0.05–2.48% of crop treated)	August-October 2004 (after flowering of *Zea mays*); no further data analysis	imi	48	0	<0.05			Nguyen et al. (2009)
	USA (Florida, California, Pennsylvania or 13 states) + outside U.S. and Canada	No data	2007/2008 Survey of 13 apiaries in Florida +California, from 47 colonies in Pennsylvania orchard and from ‘other’ samples; no further data analysis	imi	140	0	<2.0		Many pesticides detected	Mullin et al. (2010)
				thc		0	<1.0			
				thm		0	<1.0			
				ace		0	<5.0			
	Greece, Peloponnesus region	No data no data	Apiaries with bees exhibiting atypical behavior; no further data analysis	imi	5	60	27	14–39	Detection limits not given	Bacandritsos et al. (2010)
				clo		0	<LOD			
				thm		0	<LOD			
				ace		0	<LOD			
Bees wax	Belgium, different areas	No data	Monitoring; no further details	imi	98	0	<0.1			Pirard et al. (2007)
	Belgium, different areas	Maize (0.05–2.48% of crop treated)	August–October 2004 (after flowering of *Zea mays*); no further data analysis	imi	48	0	<0.05			Nguyen et al. (2009)
	USA (Florida, California, Pennsylvania or 13 states) + outside USA and Canada	No data	2007/2008 Survey of 13 apiaries in Florida + California; from 47 colonies in Pennsylvania orchard; from ‘other’ samples; no further data analysis	imi	208	0.96	–	2.4–13.6	Many pesticides detected	Mullin et al. (2010)
				thc		1.9	0.1	<1.0–8.0		
				thm		0	<1.0			
				ace		0	<5.0			
	Guelph, Canada	*Brassica napus*	1 ha fields; treated 400 g AI kg^−1^ seed (= 32 g ha^−1^) +control; 4 bee colonies per field for 21-d; sampling at 14-d intervals; no further data analysis	clo	No data	Few	–	<0.5 (treated and control)		Cutler and Scott-Dupree (2007)

*ace* acetamiprid, *clo* clothianidin, *imi* imidacloprid, *thc* thiacloprid, *thm* thiametoxam

^a^When no residues are detected, the limit of detection (LOD) is given

^b^Only one sample was positive

Several studies were performed across Europe as well as North America (one study). Some studies involved a large scale analysis of samples collected over an extended area and in different years (Genersch et al. 2010; Chauzat et al. 2011), while others did a more or less nation-wide survey in one or two sampling years (Pirard et al. 2007; Nguyen et al. 2009; Bernal et al. 2010; Garcia-Chao et al. 2010; Mullin et al. 2010). A few studies focused on a limited number of samples (Bacandritsos et al. 2010) or did not mention the number of samples analyzed (Cutler and Scott-Dupree 2007). In some studies, a wide range of pesticides was measured in different bee-related products (Bernal et al. 2010; Chauzat et al. 2009; Mullin et al. 2010; Genersch et al. 2010), while others solely focused on neonicotinoid pesticides. Only few studies did include the analysis of metabolites.

An extensive inventory of imidacloprid in bee-collected pollen, honey and bees was performed by Chauzat et al. (2006, 2009, 2011), involving five sites across France with sampling of bee hives of five beekeepers in each area for 3 years and with four sampling events per year. Imidacloprid was found in 40.5 and 21.8% of the pollen and honey samples, respectively. The metabolite 6-chloronicotinic acid was present in 33.0 and 17.6% of the respective samples. The sampling took place in four agricultural areas and one natural area. Using a χ^2^ test, frequency of imidacloprid + metabolite detection in pollen was shown to be significantly higher in 2003 compared to 2005; there was no difference for honey samples (Chauzat et al. 2011). No significance difference was found in the frequency of pesticide residue detection in pollen and honey between the different sampling areas (Chauzat et al. 2006, 2009). It is not known at what scale imidacloprid was applied in the agricultural areas where sampling took place. Neither is known what were the main plant species represented by the pollen samples collected.

As presented in Table 1, the average imidacloprid residue levels in positive pollen samples ranged between 0.9 and 3.1 μg kg^−1^, while levels in honey and beeswax were generally lower. Concentrations of 6-chloronicotinic acid were only exceeding the limit of detection in the studies of Chauzat et al. (2006, 2009, 2011), with average concentrations of 1.2 (>0.3–9.3) μg kg^−1^ and 1.2 (>0.3–10.2) μg kg^−1^ in pollen and honey, respectively. Other studies reported in general lower frequencies of imidacloprid presence in pollen, honey and beeswax samples. Nguyen et al. (2009), who sampled in an area with 13.2% of the maize crop receiving seed dressing, detected imidacloprid in 8.4% of the honey samples, but levels were always below the limit of quantification (0.5 μg kg^−1^). In a study in northern America, thiacloprid and acetamiprid were present in 5.4% of the pollen samples, while thiacloprid was also measured in 1.9% of the beeswax samples (Mullin et al. 2010). Also in Germany, thiacloprid was the most abundant neonicotinoid as it was detected in 33% of the pollen samples at concentration levels up to 199 μg kg^−1^ (Genersch et al. 2010) (Table 1). In pollen collected at 1 and 6 days after spraying of apple trees in Slovenia with Calypso 480 SC at a dose of 0.2 kg ha^−1^ (approximately 0.1 kg AI ha^−1^), respective thiacloprid levels of 60 and 30 μg kg^−1^ were recorded. In bee bread, no thiacloprid was detected (detection limit 10 μg kg^−1^) (Smodis Skerl et al. 2009).

The best measure of exposure and bioavailability are concentrations in honey bees. The study of Chauzat et al. (2011) found imidacloprid in 11.2% of the honey bee samples, while the main metabolite 6-chloronicotinic acid was detected in 18.7% of the samples. Average concentrations were 1.2 (>0.3–11.1) and 1.0 (>0.3–1.7) μg kg^−1^, respectively. Also for honey bees, there were no significant seasonal and geographic differences in the frequencies of imidacloprid or 6-chloronicotinic acid residue detection (Chauzat et al. 2011). For honey bees, other studies did not detect imidacloprid in the bees. Only in the study of Bacandritsos et al. (2010) higher imidacloprid concentrations were measured in honey bees. This study however, concerned only five samples. As shown in Table 1, no other neonicotinoid insecticides were detected in honey bees in the other inventories performed across Europe and North America.

The low residue levels in honey bees probably are best explained from the fast imidacloprid metabolism by the honey bee *A. mellifera*. After exposure to sugar water dosed at 20, 50 or 100 μg ^14^C-imidacloprid kg^−1^ honey bee, half-lives were 4–5 h (Suchail et al. 2004a, b). The major metabolites are 4- and 5-hydroxy-imidacloprid and olefin. Olefin peaked after about 4 h, while the hydroxy metabolite(s) appeared either immediately after termination of exposure and then decreased in concentration (Suchail et al. 2004b) or showed a peak after about 4 h (Suchail et al. 2004a). The total amount of imidacloprid and metabolites in honey bees decreased with a half-life of 25 h (Suchail et al. 2004a). Imidacloprid was the main compound in the abdomen (38% of accumulated ^14^C) directly after treatment. In the head, four metabolites were detected with imidacloprid levels always being ≤5% of the ingested dose, and olefin and 4- and 5-hydroxy-imidacloprid being the main metabolites after 24 and 30 h, respectively. Imidacloprid and its metabolites were also detected in other body parts of the honey bee (hemolymph, midgut, rectum) with highest amounts in the thorax (Suchail et al. 2004a). It should be noted that dosages applied in these metabolism studies are much higher than the levels found in the field and might even be in the toxic range. The relevance of these data for the metabolism at field-realistic concentrations therefore remains uncertain.

Acetamiprid was also rapidly metabolized in bees, with a half-life of 25 min after oral administration with sugar water (100 μg kg^−1^) and producing four metabolites. The major metabolite had a peak corresponding to approximately 48% of the dose after 8 h, and the other three metabolites reached maximum levels of 22–25%. After 72 h, the bees contained only metabolites. The metabolism of ^14^C-acetamiprid seems to be tissue specific and showed a similar distribution pattern in the honey bee as imidacloprid (Brunet et al. 2005).

## Side-effects of neonicotinoid insecticides in bees

### Acute lethal toxicity

To date the evaluation of potential risks of insecticides is directed by guidelines like the Directive 91/414 in Europe and the Federal Insecticide, Fungicide and Rodenticide Act in the USA. Measurements of lethal toxicity are conducted by scoring the numbers of dead bees after 24–48 h and then the corresponding median lethal dose/concentration (LD_50_ and/or LC_50_) is calculated. Tables 2 and 3 give an overview of the reported acute LD_50_ and LC_50_ values for neonicotinoid insecticides at the individual (organism) level. Based on this it is clear that several factors play a role:

**Table 2:** Overview of the lethal and sublethal side-effects by imidacloprid to individual (organism level) honey bees (*A. mellifera*), bumble bees (*B. impatiens*) and solitary bees as determined in different studies by oral/contact exposure under laboratory and (semi-)field conditions

Species	Exposure	Side-effects	References
*A. mellifera*	Lab + contact: acute (no information on concentration range), individual bees	LD_50_-24 h: 18 ng bee^−1^	Iwasa et al. (2004)
*A. mellifera*	Lab + oral: acute exposure to 0.12 and 12 ng bee^−1^	Reduction of associative learning at 12 ng bee^−1^	Decourtye et al. (2004a, b)
*A. mellifera*	Lab + semi-field: oral exposure: 24 μg kg^−1^ in syrup	Decreased foraging activity on the food source and on the hive entrance; effect on the learning performance	Decourtye et al. (2004a, b)
*A. mellifera*	Lab + oral: acute exposure to 0.2–3.2 mg l^−1^	LD_50_-48 h: 30 ng bee^−1^	Decourtye et al. (2003)
*A. mellifera*	Lab + oral: chronic exposure (no information on concentration)	LOEC on survival of winter bees: 24 μg kg^−1^ LOEC on associative learning via PER assay on winter bees (12 μg kg^−1^) and summer bees (12 μg kg^−1^)	Decourtye et al. (2003)
*A. mellifera*	Lab + contact: acute exposure to 1.25–20 ng bee^−1^	LOEC for PER habituation: 1.25 ng bee^−1^ LOEC for mobility: 1.25 ng bee^−1^; mobility reduced at 2.5–20 ng bee^−1^	Lambin et al. (2001)
*A. mellifera*	Lab + oral: acute exposure to 0.1 and 81 ng bee^−1^	LD_50_-48 h: between 41 and >81 ng bee^−1^; NOED: ≤1.25 ng bee^−1^; reduced sucrose uptake by 33% at 81 ng bee^−1^	Nauen et al. (2001)
*A. mellifera*	Lab + contact: acute exposure to 40–154 ng bee^−1^	LD_50_-48 h: between 49 and 104 ng bee^−1^	Nauen et al. (2001)
*A. mellifera*	Lab + oral: acute exposure to 0.7 mg seed^−1^	LD_50_-48 h: 4–41 ng bee^−1^	Schmuck et al. (2001)
*A. mellifera*	Lab + oral: chronic exposure (39 days) to sunflower nectar contaminated with 0.002–0.02 μg kg^−1^	NOEC for mortality, feeding activity, wax comb production, breeding performance and colony vitality: 0.02 μg kg^−1^	Schmuck et al. (2001)
*A. mellifera*	Summary of data of more than 30 lab and (semi-)field tests	Repellent antifeedant effect at 500–1,000 μg l^−1^ No adverse effects expected at residue levels of <20 μg l^−1^	Maus et al. (2003)
*A. mellifera*	Field + oral: chronic exposure	NOEC for the intraspecific communication: 10 μg l^−1^ NOEC for survival, foraging activity, colony development, brood status and changes in pollen/nectar stores: 20 μg l^−1^	Kirchner (1999)
*A. mellifera*	Lab + oral: acute exposure to 10–10,000 μg l^−1^	LD_50_-48 h: 60 ng bee^−1^	Suchail et al. (2001)
*A. mellifera*	Lab + oral: young bees chronically (10 days) exposed to 0.1, 1 and 10 μg l^−1^	50% Mortality	Suchail et al. (2001)
*B. impatiens*	Lab + contact: acute exposure via a Potter spray tower (no information on concentration range)	LC_50_-48 h: 322 mg l^−1^	Scott-Dupree et al. (2009)
*O. lignaria*		LC_50_-48 h: 7 mg l^−1^	
*M. rotundata*		LC_50_-48 h: 17 mg l^−1^	

*NOEC* no-observed effect concentration, *NOED* no-observed effect dose, *LOEC* lowest observed effect concentration, *PER* proboscis extension reflex

**Table 3:** Lethal effect concentrations of neonicotinoids for workers of the honey bee (*A. mellifera*) by oral and contact exposure as determined in different laboratory studies

Neonicotinoid	Exposure	LD_50_ (μg bee^−1^)	References
Parent compound			
Acetamiprid	Contact: individual bee (acute; no info on concentration range)	24 h: 7.07	Iwasa et al. (2004)
Acetamiprid	Contact + oral: individual bee (acute; no information on concentration range)	48 h: 14.5 (oral) + 8.09 (contact)	Decourtye and Devillers (2010)
Acetamiprid	Contact to dry residue + oral: 100 mg l^−1^ (acute; 2 days exposure for contact and 3 days for oral exposure)	Harmless	Laurino et al. (2011)
Clothianidin	Contact: individual bee (acute; no information on concentration range)	24 h: 0.022	Iwasa et al. (2004)
Clothianidin	Contact + oral: individual bee (acute; no information on concentration range)	48 h: 0.044 (contact) +0.003 (oral)	Decourtye and Devillers (2010)
	Contact to dry residue: 75–1.5 mg l^−1^ (2 days exposure) Oral: 75 mg l^−1^ to 7.5 μg l^−1^ (acute; 3 days)	No LD_50_ determined but 100% loss at 15 mg l^−1^ after 48 h 48 h: 0.003 μg l^−1^	Laurino et al. (2011)
Dinotefuran	Contact: individual bee (acute; no information on concentration range)	24 h: 0.075	Iwasa et al. (2004)
Dinotefuran	Oral: individual bee (acute; no information on concentration range)	48 h: 0.023	Decourtye and Devillers (2010)
Nitenpyram	Contact: individual bees (acute; no information on concentration range)	24 h: 0.138	Iwasa et al. (2004)
Thiacloprid	Contact: individual bees (acute; no information on concentration range)	24 h: 14.6	Iwasa et al. (2004)
Thiacloprid	Contact: individual bees (acute; no information on concentration range)	24 h: 24.2	Elbert et al. (2000)
Thiacloprid	Contact + oral: individual bees (acute; no information on concentration range)	48 h: 38.8 (contact) + 17.3 (oral)	Decourtye and Devillers (2010)
Thiacloprid	Contact dry residue + oral: 144 mg l^−1^ (acute; 2 days exposure for contact)	Harmless	Laurino et al. (2011)
Thiamethoxam	Contact: individual bees (acute; no information on concentration range)	24 h: 0.03	Iwasa et al. (2004)
Thiamethoxam	Contact + oral: individual bees (acute; no information on concentration range)	48 h: 0.024 (contact) + 0.005 (oral)	Decourtye and Devillers (2010)
Thiamethoxam	Contact dry residue: 100–1 mg l^−1^ (2 days exposure)	No LD_50_ determined but 100% loss at 100 mg l^−1^ after 6 h	Laurino et al. (2011)
Thiamethoxam	Oral: 100 mg l^−1^ to 10 μg l^−1^ (3 days exposure)	48 h: 0.004 μg l^−1^	Laurino et al. (2011)
Metabolite (parent compound)			
* N*-demethyl acetamiprid (acetamiprid)	Contact: individual bees (acute; no information on concentration range)	24 h: >50	Iwasa et al. (2004)
6-Chloro-pyridilmethyl alcohol (acetamiprid)	Contact: individual bees (acute; no information on concentration range)	24 h: >50	Iwasa et al. (2004)
6-Chloro-nicotinic acid (acetamiprid)	Contact: individual bees (acute; no information on concentration range)	24 h: >50	Iwasa et al. (2004)
Oleofin (imidacloprid)	Oral: acute (no information on concentration range)	48 h: >0.036	Nauen et al. (2001)
Oleofin (imidacloprid)	Oral: 10–10,000 μg kg^−1^ (acute) Oral: 0.1–10 μg l^−1^ (chronic: 10 days)	48 h: 0.028 (acute) no LD_50_ determined (chronic) but 30% mortality with 1 μg l^−1^ after 125 h	Suchail et al. (2001)
5-OH-imidacloprid (imidacloprid)	Oral: acute (no information on concentration range)	48 h: 0.159	Nauen et al. (2001)
5-OH-imidacloprid (imidacloprid)	Oral: 1.25–20 mg l^−1^ (acute)	48 h: 0.153	Decourtye et al. (2003)
5-OH-imidacloprid (imidacloprid)	Oral: 10–10,000 μg kg^−1^ (acute) Oral: 0.1–10 μg l^−1^ (chronic: 10 days)	48 h: 0.258 (acute) no LD_50_ determined (chronic) but 40% mortality with 1 μg l^−1^ after 125 h	Suchail et al. (2001)
Di-OH-imidacloprid (imidacloprid)	Oral: acute (no information on concentration range)	48 h: >0.049	Nauen et al. (2001)
Urea-metabolite (imidacloprid)	Oral: acute (no information on concentration range)	48 h: >100	Nauen et al. (2001)
6-Chloronicotinic acid (imidacloprid)	Oral: acute (no information on concentration range)	48 h: >122	Nauen et al. (2001)

Toxicity is dependent on the route of exposure with contact being less toxic than oral. The oral LD_50_s, however, showed large variability over the different studies with neonicotinoids (Decourtye and Devillers 2010; Laurino et al. 2011). The process of trophallaxis may have contributed to differences in the uptake and accumulation of insecticide among the worker bees, and high imidacloprid doses may cause a reduction of sugar water consumption (Nauen et al. 2001).

Upon topical treatment, nitro-containing neonicotinoids (imidacloprid, clothianidin, thiamethoxam, nitenpyram and dinotefuran) were more toxic than the cyano-group containing ones (acetamiprid and thiacloprid) (Iwasa et al. 2004; Laurino et al. 2011). A similar high toxicity of imidacloprid and thiamethoxam was also found for the bumble bee *Bombus terrestris* (Mommaerts et al. 2010). The lower toxicity of the cyano-group neonicotinoids can be attributed to their fast biotransformation (Suchail et al. 2004a, b; Brunet et al. 2005) and the existence of different nAChR subtypes (Jones et al. 2006). For contact exposure Iwasa et al. (2004) ranked the neonicotinoid insecticides based on their 24-h LD_50_ as follows: for the nitro-group: imidacloprid (18 ng bee^−1^) > clothianidin (22 ng bee^−1^) > thiamethoxam (30 ng bee^−1^) > dinotefuran (75 ng bee^−1^) > nitenpyram (138 ng bee^−1^); and for the cyano-group: acetamiprid (7 μg bee^−1^) > thiacloprid (15 μg bee^−1^).

Metabolites of neonicotinoids were shown to contribute to the toxicity (Table 3) (Nauen et al. 2001, 2003; Suchail et al. 2001; Decourtye et al. 2003) except for acetamiprid with none of the metabolites being toxic (Iwasa et al. 2004). So far, most studies were conducted on metabolites of imidacloprid: those with a nitroguanidine-group (oleofin-, hydroxy-, and dihydroxy-imidacloprid) were more toxic (oral LD_50_) compared to the urea-metabolite and 6-chloronicotinic acid (Nauen et al. 2001). The metabolite of thiamethoxam, clothianidin was highly toxic for bees (Nauen et al. 2003).

For imidacloprid the toxicity varied upon insect-related factors such as the age of the bee, the colony, the subspecies used (Suchail et al. 2000, 2001; Nauen et al. 2001; Guez et al. 2003) and the health of the bees with sub-optimal protein feeding (Wehling et al. 2009) or *Nosema ceranae* infestation (Alaux et al. 2010; Vidau et al. 2011) making the bees more sensitive. Stark et al. (1995) found no effect of bee genera as the 24-h-contact LD_50_s for imidacloprid were similar in both social bees (*A. mellifera*) and solitary bees (*Megachile rotundata* and *Nomia melanderi*) (Table 2). Similar conclusions were also drawn for thiamethoxam with an LD_50_ of 30 ng bee^−1^ for *A. mellifera* and 33 ng bee^−1^ for *B. terrestris* (Iwasa et al. 2004; Mommaerts et al. 2010). Scott-Dupree et al. (2009), however, found that bumble bees (*Bombus impatiens*) were more tolerant to clothianidin and imidacloprid than *Osmia lignaria* and *M. rotundata*.

### Chronic lethal toxicity

Chronic oral/contact exposure during 10–11 days to 1 μg bee^−1^ acetamiprid and 1 ng bee^−1^ thiamethoxam caused no significant worker mortality (Aliouane et al. 2009). For imidacloprid, laboratory tests showed high worker loss when honey bees consumed contaminated pollen (40 μg kg^−1^) (Decourtye et al. 2001, 2003) and sugar water (0.1, 1.0 and 10 μg l^−1^) (Suchail et al. 2001). These results were in disagreement with field studies. Schmuck et al. (2001) reported no increased worker mortality when honey bee hives were exposed during 39 days to sunflower nectar contaminated with imidacloprid in a range of 2.0–20 μg kg^−1^. Also Faucon et al. (2005) and Cresswell (2011) concluded that oral exposure to food contaminated with imidacloprid at realistic field concentrations did not result in worker mortality. A possible explanation for this discrepancy between laboratory and field studies may be differences in experimental methodology. Indeed the toxic effect on an individual may depend on its initial physiological state and on the longevity of nest mates (Decourtye and Devillers 2010). In addition, the social interaction should be taken into consideration with exposure of honey bees over a longer period. For bumble bees the chronic toxicity of compounds (exposure time up to 11 weeks) can be determined using micro-colonies (Mommaerts and Smagghe 2011).

### Sublethal effects on reproduction

Reproduction is an important process to assure the further existence of the colony. Indeed, a loss of reproduction (brood) might be more detrimental for the colony than the loss of older bees (foragers) (Decourtye and Devillers 2010). This is further supported by studies on the division of tasks in bee colonies. For example in bumble bees (*B. impatiens*) task division is a dynamic process (weak task specialization) and so workers perform multiple tasks during their lifespan (Jandt and Dornhaus 2009). Therefore it is not unlikely that foragers are replaced by other bees when enough nurses are present in the hive. A few studies have demonstrated the adverse effects on larval development following exposure to imidacloprid (Tasei et al. 2000, 2001; Decourtye et al. 2005; Abbott et al. 2008; Gregorc and Ellis 2011). Decourtye et al. (2005) reported a delay in the time needed for honey bee larvae to hatch or develop as an adult when fed with food contaminated with imidacloprid at 5 μg kg^−1^. Similar observations were also made by Abbott et al. (2008) for *O. lignaria* when imidacloprid was dosed at 30–300 μg kg^−1^ food. Also for bumble bees (*B. terrestris*) a reduction of the brood (larvae) was seen in micro-colonies orally exposed to contaminated sugar water (10 μg kg^−1^ imidacloprid) + pollen (6 μg kg^−1^ imidacloprid) (Tasei et al. 2000) (Table 4).

**Table 4:** Overview of the concentrations of imidacloprid causing lethal and sublethal effects on (micro-)colony level in honey bees (*A. mellifera*) and bumble bees (*B. terrestris*, *B. impatiens*) as determined in different studies by oral/contact exposure under laboratory and (semi-)field conditions

Species	Exposure	Toxicity	References
*A. mellifera*	Field + oral: 0.5–5 μg l^−1^ in syrup (chronic)	NOEC for survival: 5 μg l^−1^ NOEC for brood, adult foraging activity, adult bee population level, number of frames with brood after overwintering and general colony vitality: 5 μg l^−1^	Faucon et al. (2005)
*A. mellifera*	Lab + oral: 100–1,000 μg l^−1^ (acute)	500–1,000 μg l^−1^: bees disappeared at the hive/feeding site up to 24 h 100 μg l^−1^: no effect on homing rate	Bortolotti et al. (2003)
*A. mellifera*	Lab + oral: 0.12 and 12 ng bee^−1^ in syrup (acute) Lab + oral: 24 μg kg^−1^ in syrup (24 h) Semi-field + oral: 24 μg kg^−1^ in syrup (24 h)	Increase of the cytochrome oxidase labeling, negative effect on the PER assay with 12 ng bee^−1^ but not with 0.12 ng bee^−1^ Negative effect on the PER assay Decreased foraging activity, hive entrance activity, sucrose consumption and brood size	Decourtye et al. (2004a, b)
*A. mellifera*	Field + oral: foraging on maize fields treated with imidacloprid (chronic; no information about the dose)	No relation between imidacloprid treated maize fields and bee mortality in apiaries	Nguyen et al. (2009)
*A. mellifera*	Semi-field + oral: 40–6,000 μg l^−1^ in syrup (acute)	LOEC for foraging behavior: 50 μg l^−1^ >1,200 μg l^−1^ abnormalities in revisiting the feeding site	Yang et al. (2008)
*A. mellifera*	Lab + oral: >100 μg kg^−1^ (chronic)	NOEC survival: 2–20 μg kg^−1^ in sunflower nectar 20 μg l^−1^: decrease in foraging activity; >100 μg l^−1^: reduce in foraging behavior for 30–60 min >50 μg l^−1^: increase in interval between successive visits at a feeder	Schmuck (1999); Schmuck et al. (2001)
*A. mellifera*	Semi-field + oral: 48 μg kg^−1^ in syrup (chronic)	Affected syrup consumption, foraging activity and brood size	Ramirez-Romero et al. (2005)
*B. terrestris*	Lab + oral: 10–25 μg kg^−1^ in syrup + 6–16 μg kg^−1^ in pollen (chronic exposure: 85 days)	Increased worker mortality after 30 days No effect on food consumption and male emergence; but 10 μg kg^−1^ in syrup + 6 μg kg^−1^ in pollen: reduction in brood size	Tasei et al. (2000)
*B. terrestris*	Field + oral: foraging on plants grown from imidacloprid-treated sunflower seeds treated with 0.7 mg seed^−1^ (chronic exposure: 9 days)	No effect on foraging and colony vitality	Tasei et al. (2001)
*B. terrestris*	Lab + oral: 200 mg l^−1^ to 10 μg l^−1^ in sugar water (chronic 11 weeks: test without foraging: test with foraging:	Test without foraging: LC_50_: 59 μg l^−1^; EC_50_: 37 μg l^−1^; NOEC for reproduction: 20 μg l^−1^ Test with foraging: LC_50_: 20 μg l^−1^; EC_50_: 3.7 μg l^−1^; NOEC for reproduction: <2.5 μg l^−1^	Mommaerts et al. (2010); Mommaerts and Smagghe (2011)
*B. terrestris*	Semi-field + oral: 20–2 μg l^−1^ in sugar water (chronic 2 weeks)	NOEC for brood, colony growth and foraging activity: 2 μg l^−1^	Mommaerts et al. (2010)
*Bombus occidentalis*, *B. impatiens*	Lab + oral: 7–30 ng g^−1^ pollen (chronic: twice weekly)	NOEC for brood, numbers of males, queens and workers, worker weight, pollen consumption, forage ability on complex artificial flowers: 7 ng g^−1^	Morandin and Winston (2003)
*B. impatiens*	Field + dry residue (acute exposure) Granules + spray: 0.45 kg ha^−1^ Spray + irrigation: 0.34 kg ha^−1^ Spray + non-irrigated: 0.34 kg ha^−1^	No effect on colony vitality and worker behaviour No effect on colony vitality, worker defensive response and on foraging preference Reduction in numbers of brood chambers, honey pots, workers, colony weight and on foraging preference	Gels et al. (2002)

*NOEC* no-observed effect concentration, *LOEC* lowest observed effect concentration, *PER* proboscis extension reflex

### Sublethal effects on behavior

Sublethal effects which interfere with the process of food collection and subsequent social colony life and pollination need to be considered (Thompson and Maus 2007; Desneux et al. 2007; Mommaerts and Smagghe 2011). Over the past years several laboratory and (semi-) field tests have been developed to investigate the effect of neonicotinoid insecticides on motor and sensory functions linked to the foraging capacity of bees.

Neonicotinoid insecticides act as neurotoxic agents and affect the mobility of bees by inducing symptoms such as knockdown, trembling, uncoordinated movements, hyperactivity and tremors (Lambin et al. 2001; Nauen et al. 2001; Suchail et al. 2001; Medrzycki et al. 2003; Colin et al. 2004). These symptoms are easy to observe at high exposure levels, while the effect of a lower dose might be more difficult to see. El Hassani et al. (2005) therefore developed a new laboratory test consisting of a plastic box with a transparent plate that was illuminated, enabling to record the vertical displacement of the bees. Contact exposure to imidacloprid at 1.25 ng bee^−1^ and to acetamiprid at ≤0.5 μg bee^−1^ increased locomotor activity, whereas imidacloprid at 2.5 ng bee^−1^ significantly decreased bee mobility (Lambin et al. 2001). No negative effects on the locomotor activity were found after acute and chronic (11 days) exposure (oral) to acetamiprid at 0.1 μg bee^−1^ and after acute exposure (contact and oral) to thiamethoxam at 1 ng bee^−1^ (El Hassani et al. 2008; Aliouane et al. 2009).

Another sublethal endpoint affected by neonicotinoids (acetamiprid and thiamethoxam) is the proboscis extension reflex (PER) following perception of sucrose and water (El Hassani et al. 2008; Aliouane et al. 2009). The effect was demonstrated to be dependent on the route, duration and dose of exposure (El Hassani et al. 2008; Aliouane et al. 2009). In addition, by conditioning of the PER using an odor, various studies demonstrated changes in the olfaction learning of bees upon exposure to neonicotinoids. Learning was reduced after chronic (up to 11 days) exposure to imidacloprid (winter bees: 48 μg kg^−1^; oral), the metabolite 5-hydroxy-imidacloprid (winter bees: 120 μg kg^−1^; oral) and thiamethoxam (0.1 ng bee^−1^; contact) (Decourtye et al. 2003; El Hassani et al. 2008; Aliouane et al. 2009). By expanding the PER test also more information was gained on how neonicotinoids interfere with the memory process. Oral uptake of 0.1 μg bee^−1^ acetamiprid induced long-term memory impairments, whereas chronic contact to 1 ng bee^−1^ thiamethoxam (corresponding with 1/5 of the LD_50_) did not cause long-term effects as recovery of memory was seen after 48 h (El Hassani et al. 2008; Aliouane et al. 2009). For imidacloprid, different authors reported on medium-term memory effects (Table 2) (Decourtye et al. 2001, 2003, 2004a; Lambin et al. 2001). Decourtye et al. (2004b) documented that such effects may result from an increase of the cytochrome oxidase activity, related with aberrations of the mushroom bodies in the brain. The effects of imidacloprid on habituation of PER depended on the age of the bees tested and thus on their task within the colony (Guez et al. 2001, 2003). Although it is obvious that neonicotinoids can interfere with the olfactory learning process in different ways, extrapolation of these laboratory effects to a real exposure situation in the field therefore is complex and difficult.

Neurotoxic compounds such as neonicotinoids were also reported to interfere with the orientation process of honey bees. Associative learning between a visual mark and a reward (sugar solution) in a complex maze showed that only 38% of the bees found the food source after oral ingestion of thiamethoxam at 3 ng bee^−1^ compared to 61% in the control group (Decourtye and Devillers 2010). In another study using marked foragers that were first trained to forage on artificial feeders, Bortolotti et al. (2003) noticed that a 500 m distance between the hive and the feeding area resulted in no foragers at the hive/feeding area up to 24 h after treatment when foragers were fed with imidacloprid at 500 and 1,000 μg l^−1^ (Table 4). The latter authors also found that a lower concentration (100 μg l^−1^ imidacloprid) caused a delay in the returning time (to hive or feeding area) of the foragers. This was confirmed by Ramirez-Romero et al. (2005) and Yang et al. (2008). Based on these results it is obvious that neonicotinoids interfere with the foraging capacity of bees. However, the different (semi-)field studies provide a mixed pallet of results. For instance, Cutler and Scott-Dupree (2007) reported no side-effects on honey bees foraging when hives were exposed to flowering canola grown from clothianidin-treated seeds. The same conclusion was drawn for imidacloprid (Schmuck et al. 2001; Faucon et al. 2005; Nguyen et al. 2009), but for thiacloprid foraging was only reduced up to 48 h after treatment (Schmuck et al. 2003). Similarly, there was no negative effect on *B. terrestris* foraging on imidacloprid- and thiamethoxam-treated plants (Colombo and Buonocore 1997; Tasei et al. 2001; Alarcón et al. 2005), and also no side-effects on *B. impatiens* exposed to weedy turf treated with imidacloprid by irrigation, to field residue levels of imidacloprid and to the highest residue level of clothianidin recovered in pollen (6 μg kg^−1^) (Gels et al. 2002; Morandin and Winston 2003; Franklin et al. 2004). It needs to be remarked that the *B. impatiens* colonies, foraging on non-irrigated imidacloprid-treated weed, showed a significant reduction in nest development (brood chambers, honey pots and worker biomass) and foraging activity (Gels et al. 2002). From these observations it is clear that there exists a discrepancy between field and laboratory tests for sublethal effects. Decourtye and Devillers (2010) documented that this was due to the ability of bees to change their behavior in response to pesticide perception. Indeed, honey bees responded by rejection when they perceived a sucrose solution contaminated with 20 μg l^−1^ imidacloprid, which resulted in a significant reduction of the foraging activity (Mayer and Lunden 1997; Kirchner 1999; Schmuck 1999; Maus et al. 2003). This protective avoidance behavior of bees towards contaminated food might reduce risk of pesticide exposure and effects. Such behavior on the other hand contributed to a decrease in general fitness of the bees with 6–20%, as deduced from statistically fitted performance data (Cresswell 2011).

It has recently been shown that bees became exposed to neonicotinoids in seed-coated fragments also via guttation fluid. After feeding on dew no honey bee mortality was observed, but feeding guttation fluid from directly treated plants did result in high mortality (Girolami et al. 2009). Also direct exposure to dust from the planting machine resulted in high bee mortality (Marzaro et al. 2011). In the latter experiments, clothianidin residues in dead bees averaged 279 ± 142 ng bee^−1^ at high humidity and 514 ± 174 ng bee^−1^ at low humidity, which by far exceed the LD_50_ of 21.8 ng bee^−1^. Similar findings were also reported by Girolami et al. (2011), exposing honey bees to dust from clothianidin and imidacloprid-treated seeds. Their study showed that mortality of exposed honey bees only occurred at high air humidity.

### Effects on overwintering of bees

During the last years a loss of overwintering bee colonies was noticed. Although identification of the causes of this disappearance is difficult, it was argued that reduced bee health might be initially caused by the chronic exposure to pesticides. So far only two studies have been conducted in this context for neonicotinoids. Using 8 honeybee colonies, Faucon et al. (2005) demonstrated that chronic exposure during the summer season (33 days) to 0.5 and 5.0 μg l^−1^ imidacloprid in saccharose syrup did not affect the overwintering abilities of honey bees. Similarly, spring assessment of colony development (brood, worker biomass and colony health) was not affected in overwintered colonies that had foraged on flowering canola grown from seed treated with clothianidin at 0.4 mg kg^−1^, representing the highest recommended rate (Cutler and Scott-Dupree 2007). In conclusion, these studies demonstrated no long-term effects on honeybee colonies of environmentally relevant concentrations.

### Mixture toxicity

This section will focus on cases in which synergistic effects were found when exposing organisms to mixtures containing neonicotinoids insecticides.

Only one study is available on the toxicity of neonicotinoids in mixtures to pollinators. Iwasa et al. (2004) found that addition of piperonyl butoxide and the fungicides triflumizole and propiconazole increased the acute toxicity (24-h LD_50_, topical application) of acetamiprid and thiacloprid to honey bees (*A. mellifera*) by factors of 6.0, 244 and 105, and 154, 1141 and 559, respectively, but had little effect on the toxicity of imidacloprid (1.5–1.9 times more toxic). The toxicity of acetamiprid was 6.3–84 times increased by the fungicides triadimefon, epoxiconazole and uniconazole-P. All synergists were topically applied at a dose of 10 μg bee^−1^ and 1 h before dosing the insecticides (Iwasa et al. 2004).

In grass shrimp larvae (*Palaemonetes pugio*) slightly synergistic effects were found when imidacloprid was applied together with atrazine (Key et al. 2007) with 96-h LC_50_ values ranging between 0.83 and 0.93 toxic units.

The toxicity of mixtures of imidacloprid and thiacloprid for earthworms (*Eisenia fetida*) was sometimes higher than expected from the toxicities of the individual chemicals. This was especially the case for earthworm weight change in a clay loam soil, where a dose-ratio dependent deviation was seen suggesting a shift from antagonism to synergism when thiacloprid accounted for more than 88% of the toxicity of the mixture (Gomez-Eyles et al. 2009). For effects on the reproduction of both nematodes (*Caenorhabditis elegans*) and daphnids (*Daphnia magna*), the mixture of imidacloprid and thiacloprid showed a dose-level dependent deviation from additivity, with synergism at low and antagonism at high exposure levels. For nematodes, the switch occurred at approximately 95% of the EC_50_ (Gomez-Eyles et al. 2009), while for daphnids this was the case at 1.5 times the EC_50_ (Pavlaki et al. 2011). Gene response profiles (transcriptomics, proteomics) in marine mollusks (*Mytilus galloprovincialis*) showed different patterns for the mixture compared to the single compounds, suggesting that the mode of action at the molecular level may be quite distinct (Dondero et al. 2010).

Synergism for effects on the population growth rate of *Ceriodaphnia dubia* was found by Chen et al. (2010) when determining the toxicity of a mixture of the nonylphenol polyethoxylate R11 and imidacloprid. Results of this study are, however, hard to interpret as only one concentration was tested. A mixture of imidacloprid with nickel showed synergistic effects on body length development of *D. magna* (Pavlaki et al. 2011).

It remains unclear how these data can be extrapolated to bee-relevant exposure situations, although it may be noted that studies of Mullin et al. (2010), Genersch et al. (2010) and Bernal et al. (2010) showed the presence of large numbers of different pesticides in bee-collected products like pollen, honey and beeswax. The data do, however, not allow for a quantitative risk analysis of possible mixture exposure.

## Risk assessment scheme for hazards by neonicotinoids in bees

A risk assessment for systemic compounds starts by identification of the exposure risk (Alix et al. 2009; Thompson 2010; Fischer and Moriarty 2011). In case exposure is likely to occur because bees are attracted to the crop and the compound can be translocated to the nectar and pollen further assessment is crucial. As given above, neonicotinoids show good systemic properties and are recovered in nectar and pollen, therefore suggesting this scheme for risk assessment can be applied for neonicotinoids.

At present Tier-1 recommends acute toxicity testing on adults and brood. However, to estimate the impact of neonicotinoids in the field a first screening should include environmental relevant doses. For neonicotinoids, contaminated food was already demonstrated to be transported to the hive where it can either be stored or used as food for larvae and adults or where it can enter the wax of the combs. In this context, Wu et al. (2001) found no larval mortality but demonstrated delayed worker development when brood was reared in highly contaminated (including low residue concentrations of several neonicotinoids) brood combs. Consequently, side-effects on brood by neonicotinoids must be assessed and no-observable effect levels (NOEL) need to be determined. When working with honey bees, care is needed as one bee gathers food and transmits it to nest mates by trophallaxis. A first study did not notice a difference between honey bees fed with imidacloprid individually or in a group as the 48-h LD_50_ of 25 ng bee^−1^ was equal for both (Decourtye and Devillers 2010). Nonetheless, future studies should give more attention to this as dilution of the product is likely to occur when food is transmitted between nest mates.

In Tier-2 the NOEL as determined under Tier-1 is used to determine the chronic oral toxicity for individual adult bees. Acute toxicity gives a first indication of the real risk but it is still an incomplete measurement. Therefore potential side-effects after long-term exposure (contact and acute) to neonicotinoids need to be evaluated. Honey bees have been exposed for a maximum of 10–11 days and 39 days in the different respective laboratory and field tests reported so far. Indeed the need for a more standardized approach on bee age, colony size and appropriate exposure was also confirmed by the Cox proportional hazard model of Dechaume-Moncharmont et al. (2003) during a 60-day dietary exposure with imidacloprid at 4 and 8 μg l^−1^. Tier-2 testing requires to consider both adult and larval stages because residues are recovered in their food, which includes pollen and nectar. Adult bees consume more nectar than pollen, while larval stages consume more pollen than nectar (Rortais et al. 2005). For the adults, a good knowledge on their foraging behavior on the crop is crucial: for instance, is the bee attracted to nectar or pollen or to both? As documented above, neonicotinoids may be translocated to both compartments of nectar and pollen, however, residue analyses so far have mainly focused on pollen. As a consequence, more data on nectar contamination need be collected since it is difficult to extrapolate toxicity data obtained with pollen to nectar. Halm et al. (2006) also confirmed the need for a better standardization of the bee categories in risk assessment as the calculated exposure to imidacloprid was higher for the group of winter bees, nectar foragers and nurses than for the group of workers and drone larvae, wax-producing bees and pollen foragers. The latter authors propose to use the predicted environmental concentration/predicted no effect concentration (PEC/PNEC) ratio approach to determine the risk instead of using LD_50_ or LC_50_ values.

Higher tier risk assessments are conducted on the colony level to include the effect of social interaction. This phase of the assessment is needed to enable drawing firm conclusions on the compatibility of the compound under field conditions. The results obtained so far for neonicotinoids (mainly for imidacloprid) under laboratory conditions do not give a good estimation of the real effect on honey bees under field conditions. Indeed honey bees only needed to use a limited number of cues in a complex maze in laboratory studies, whereas visual learning in the field is more complex. Yang et al. (2008) reported on the use of foraging bees that have been trained prior to the risk assessment test, however, the marking is very labor intensive. Alternatively, Decourtye et al. (2011) connected a microchip to the honey bee body to assess sublethal effects on the number of foraging trips by low concentrations of fipronil. For bumble bees specifically, Mommaerts et al. (2010) developed a “foraging behavior” bioassay that allows to assess in the laboratory the sublethal effects on foraging by imidacloprid, as observed in free-flying bumble bee workers in the greenhouse.

As already mentioned at Tier-1, further improvement of the reliability will be obtained when tests can be performed with environmentally relevant concentrations. The field risk assessment studies should cover all potential routes of exposure. Exposure to neonicotinoids in dust from the planting machine has been reported to result in high bee mortality, especially at high air humidities (Girolami et al. 2011; Marzaro et al. 2011). Further, exposure might also occur via the ingestion of contaminated guttation fluid. Although this route of exposure has been considered important, the data so far are not clear. As Thompson (2010) reported, the liquid is mainly present early in the morning and it remains unclear whether that corresponds to the time when bees or other pollinators are active and to what extent they ingest this fluid. In addition, it is not clear whether residues after drying of the liquid on the leaves remain a source of exposure (Thompson 2010). Tapparo et al. (2011) also reported that imidacloprid concentrations in guttation fluid did show a clear correlation with the dose applied to the seeds. Therefore, as long as no firm conclusion can be drawn, it is advisable to include this route of exposure into a risk assessment scheme for neonicotinoids.

In conclusion, assessment of risks for side-effects by use of field trials remains the final step as the field is a complex environment in which different factors may influence neonicotinoid toxicity. Concerning the effect of social interaction it needs to be remarked that for other non-*Apis* genera such as bumble bees potential side-effects on colony level can be evaluated earlier in the risk assessment, namely under Tier-2. Indeed, a standardized test with micro-colonies allows evaluating lethal and sublethal effects of neonicotinoids on bee reproduction and behavior. Micro-colonies are nests made of 3–5 new-born workers (the same age). Then, after 1 week one worker becomes dominant, like a queen in greenhouse colonies, and starts laying unfertilized eggs that develop into males while the other workers take care of the brood and forage for food. The dominant worker functions as a pseudo queen and the others as nurses and foragers. Food consists of commercial sugar water and pollen. Subsequently, the impact of neonicotinoids can be tested via different routes of exposure, namely contact exposure and orally via the drinking of treated sugar water and by eating treated pollen for 7 weeks. Other advantages of this method are the low cost, the ease of use, the possibility to work with standardized protocols and with multiple replicates resulting in sufficient statistical power to obtain reproducible data. The experimental set-up also allows social interaction to take place. Lethal effects are evaluated by scoring the number of dead workers per nest while evaluation of sublethal effects occurs by scoring the presence of honey pots, the number of dead larvae and the number of males produced per nest (Mommaerts et al. 2006a, b; Besard et al. 2011). Based on the latter endpoints, Mommaerts et al. (2010) could determine that the NOEC values for imidacloprid using such micro-colonies were equal to those obtained when using queenright colonies in the greenhouse test.

## Conclusions and targets for research and recommendations

Neonicotinoids are an important group of insecticides effective in the control of economically important pests such as aphids, leafhoppers and whiteflies. The wide application of these insecticides with a worldwide annual market of $1 billion is attributed to their selective mode of action at low doses (Aliouane et al. 2009). Neonicotinoids act as neurotoxins on the insect nervous system by interaction with the insect nAChR. In order to identify potential hazards of neonicotinoids to bees this study summarized all available data.

Via the plant sap transport neonicotinoids are translocated to different plant parts. In general, the few reported residue levels of neonicotinoids in nectar (average of 2 μg kg^−1^) and pollen (average of 3 μg kg^−1^) were below the acute and chronic toxicity levels; however, there is a lack of reliable data as analyses are performed near the detection limit. Similarly, also the levels in bee-collected pollen, in bees and bee products were low. But before drawing a conclusion, it is strongly encouraged to conduct more studies as so far only a few large studies have been undertaken in apiaries in France, Germany and North America. Moreover, the wide and increasing application of neonicotinoids in pest control will likely cause an accumulation of neonicotinoids in the environment in the future.

Many lethal and sublethal effects of neonicotinoid insecticides on bees have been described in laboratory studies, however, no effects were observed in field studies with field-realistic dosages.

The risk assessment scheme for soil-applied systemic pesticides proposed by Alix et al. (2009) and Thompson (2010) seems adequate for assessing the risks of side-effects by neonicotinoids as it takes into account the effect on different stages (adult versus larvae) and on different levels of biological organization (organism versus colony). Nevertheless, there is still a need for testing field-realistic concentrations at relevant exposure and durations and, especially for honey bees, to continue side-effect evaluation over winter and the next year in spring. The scoring of sublethal effects related to foraging behavior and learning/memory abilities, however, is very difficult. As the genomes of honey bees (*A. mellifera*) and bumble bees (*B. terrestris*, *B. impatiens*) are available, these may help to better understand the complex (network) mechanisms under natural conditions in bees. Then, treatment with pesticides like neonicotinoids will indicate which effects and responses take place at the molecular level and can be related to the exposure. A good example is the availability of a microarray of the brain of honeybees (Alaux et al. 2009). After validation, such gene/transcriptome responses can be employed as molecular ecotoxicological markers, which in turn can improve risk assessment. These molecular markers can be complementary to the robust classical endpoints of mortality and reproduction, which are assessed using individual insects and (micro-)colonies in accordance with the tier-level. These new molecular insights can also contribute to better understanding the mechanisms of action of neonicotinoids like their interaction with different nAChR in bees, also in relation to their pharmacokinetics and metabolism. The newer and safer neonicotinoids, e.g. using the cyano-group instead of the nitro-group, are good examples for further development of environmentally safer compounds employing the existence of different nAChRs in the insect nervous system. The toxicity of neonicotinoids may, however, increase by synergistic effects with other compounds as was demonstrated by Iwasa et al. (2004) for mixtures containing a cyano-group neonicotinoid. Therefore, screening for safer compounds should also include gathering more information on potential synergistic effects of mixtures containing neonicotinoids as this is currently lacking.

Finally, during the preparation of this review it was observed that results/data on concentrations, side-effects and risk assessment studies are available, but that many data are scattered and/or not publicly available. A better communication between industry, academia and government may help for a “better” risk assessment. The latter can also help to provide answers to the questions/concerns as present in the public media/society.
